# Supplementary material for: Beyond the microcirculation: sequestration of infected red blood cells and reduced flow in large draining veins in experimental cerebral malaria
Source: Nat Commun. 2024 Mar 16;15:2396. doi: 10.1038/s41467-024-46617-w (PMC10944460; doi:10.1038/s41467-024-46617-w)
Supplement: Supplementary file 1 — Supplementary Information [file 41467_2024_46617_MOESM1_ESM.pdf]

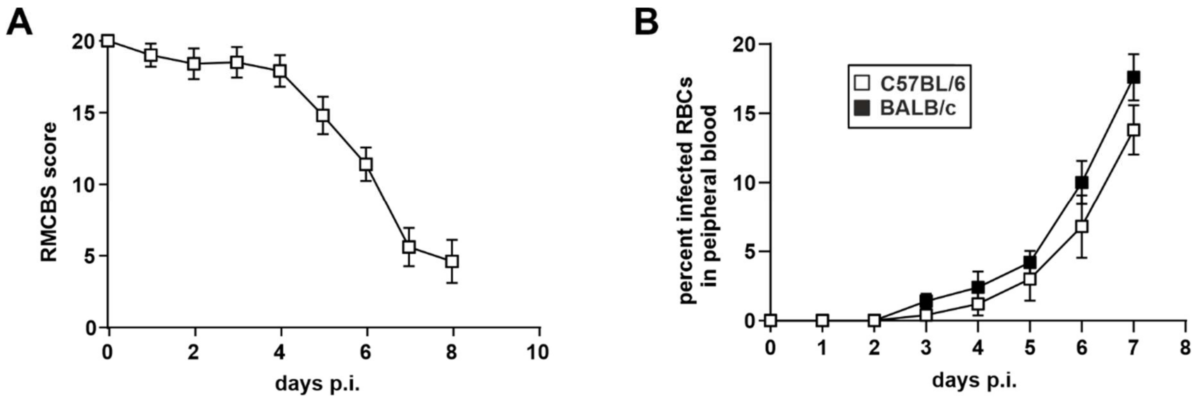

Figure S1. Progression of ECM.

(A) The RMCBS score of appearance of neurological symptoms of ECM were monitored daily up to day 8 p.i. Data represent one of two independent experiments (n=10 mice per group, 5 males and 5 females 10 weeks old). (B) The percentage of parasitized erythrocytes in the peripheral blood was enumerated daily up to day 10 p.i. by microscopic examination of Diff. Quik-stained thin blood smears. n=5 independent animals per group.

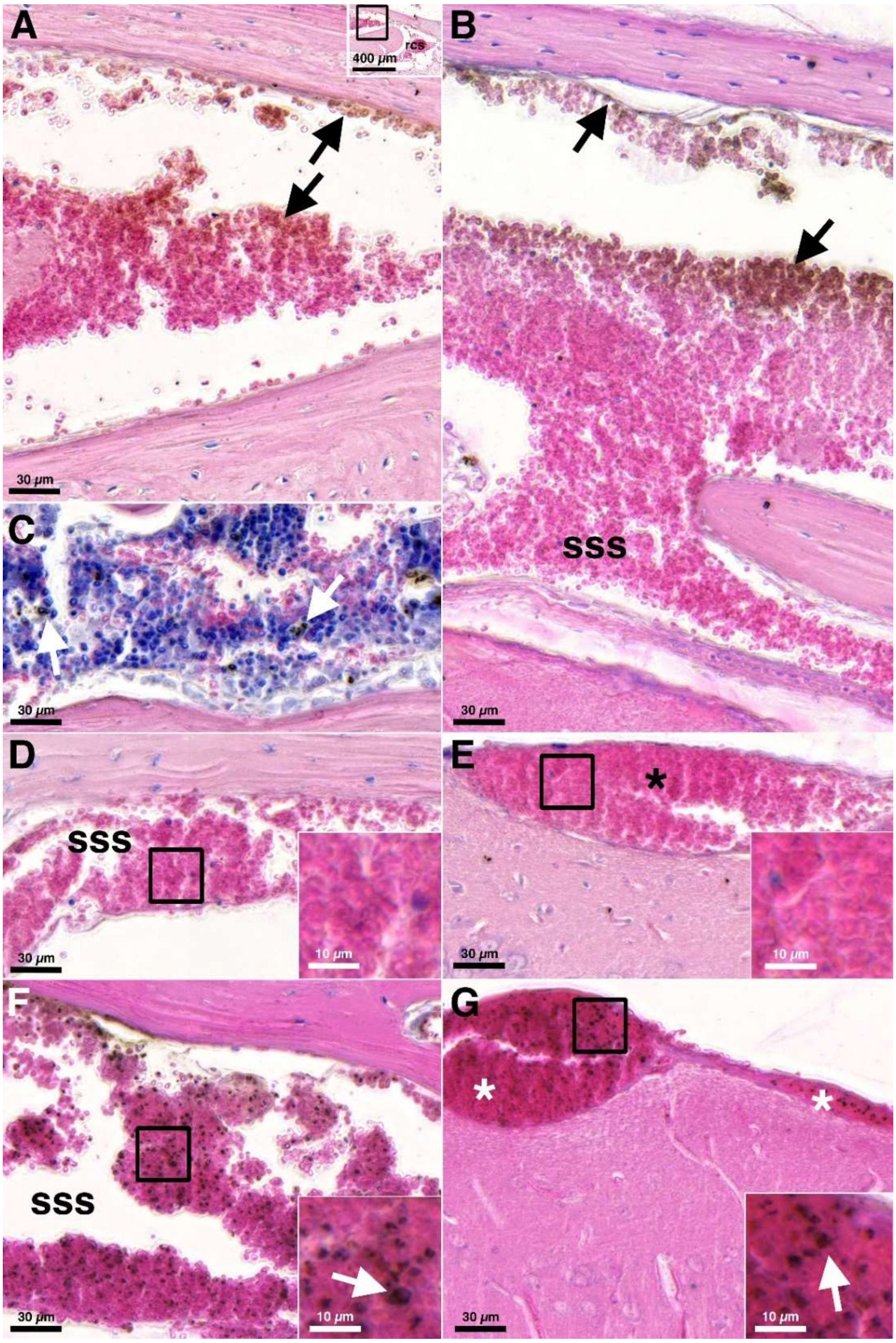

Figure S2. *Pb*NK65-infected C57BL/6 wt mice lack large deposits in the draining veins.

Giemsa-stained sagittal sections at the level of the sss caudal to the rostral confluence of the sinus (rcs). Images A to E are from *Pb*NK65, F and G from *Pb*A-infected mice at day 7 p.i. In the compartment above the sss, *Pb*NK65 infected mice show dark brown stained RBCs in similar spatial distributions in different animals (arrows in A, B). The skull bone marrow contains deposits (arrows in C). *Pb*NK65-infected mice entirely lack the large deposits present in *Pb*A-infected mice. The sss from a *Pb*A mouse is shown in F, two veins on the surface of cerebral cortex are shown in G (asterisks). Arrows in the insets point to large deposits. Note also the black-brown staining of the vessel wall of the sss (black arrowhead in F). The sss from two *Pb*NK65 infected mice are shown in (B, D), a cortical surface vein in E.

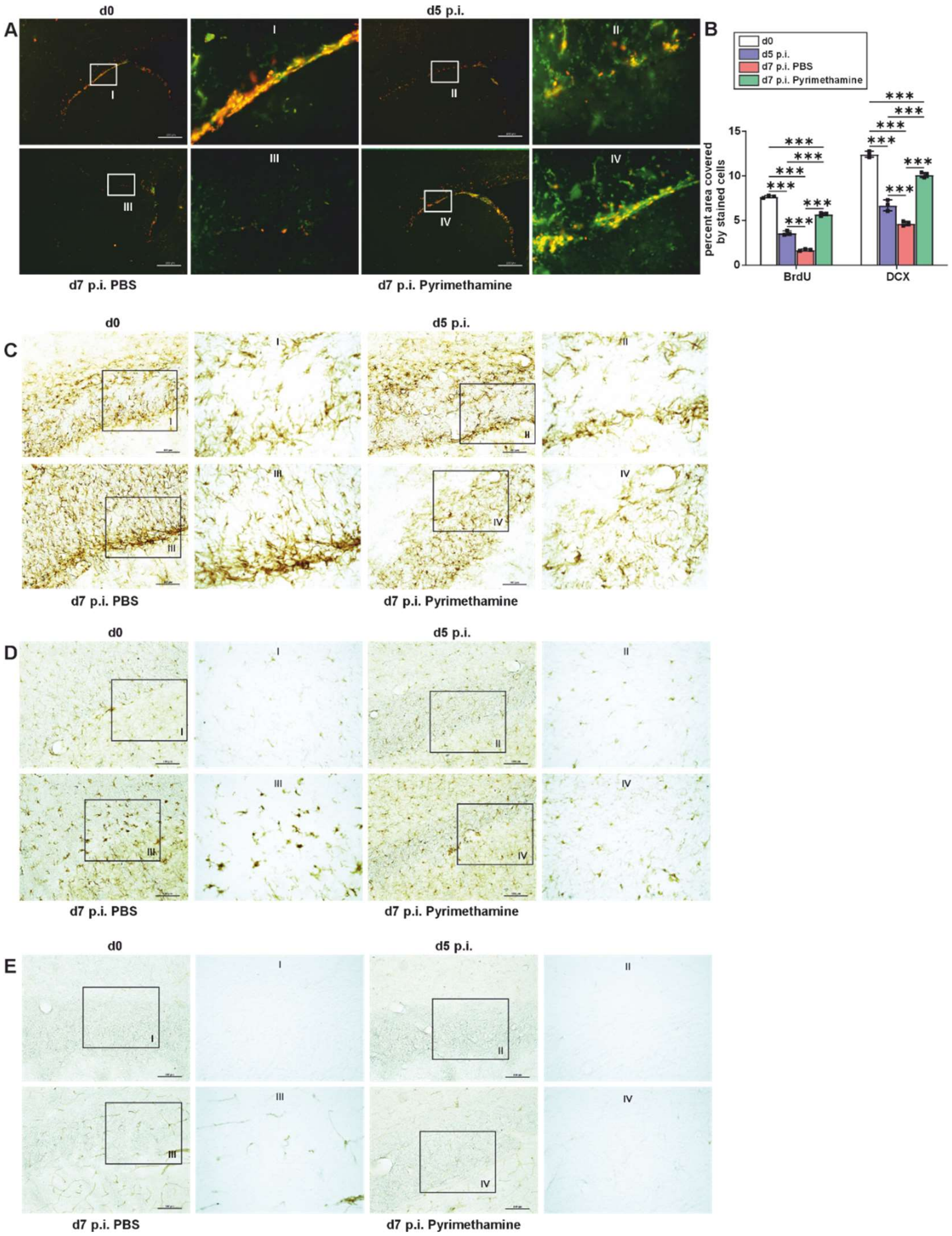

Figure S3. Progression of ECM results in disruption of the rostral migratory stream and impaired neurogenesis.

(A, B) BrdU was injected and 48 h later brains were isolated and the expression of BrdU and doublecortin (DCX) labelled neurons was analysed by immunofluorescence. Histopathology was performed on brains isolated from 10 weeks old female uninfected mice (day 0), day 5 p.i. and day 7 p.i and pyrimethamine treated infected mice on day 7 p.i.

(C-E) Increased activation of astrocytes (GFAP) (C) microglia (Iba-1) (D) and endothelial cells (CD31) (E) in the RMS region of the untreated mice at day 7 p.i. and complete recovery after anti-malarial treatment. n=3 independent animals per group for all. *, $p<0.05$; **, $p<0.01$; ***, $p<0.001$ (two-way ANOVA).

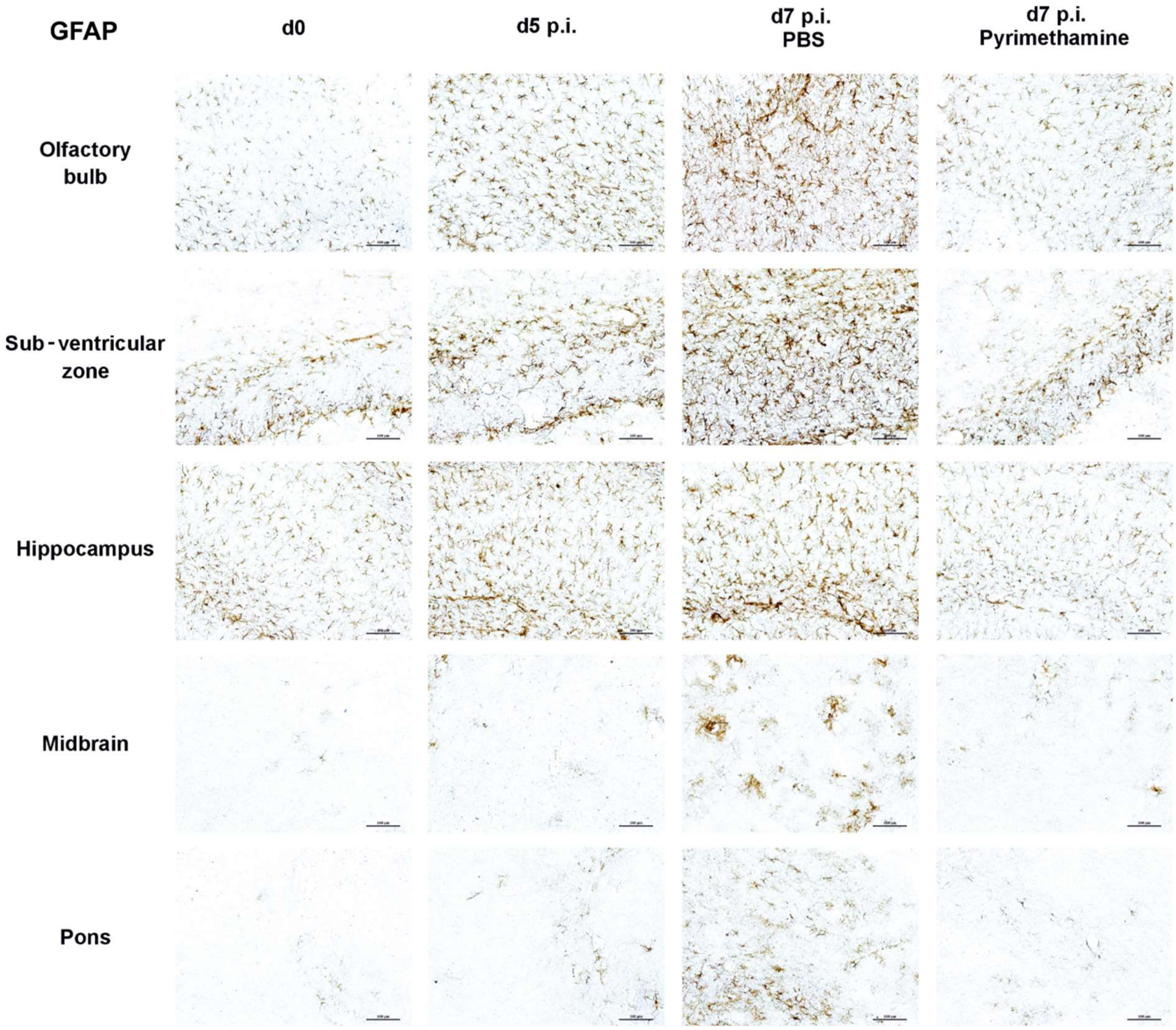

Fig S4: Immunohistochemistry to detect activation of astrocytes over the course of *Plasmodium berghei* infection and after pyrimethamine treatment. Shown are representative images of histological sections of the indicated brain regions stained with an antibody against glial fibrillary acidic protein (GFAP).

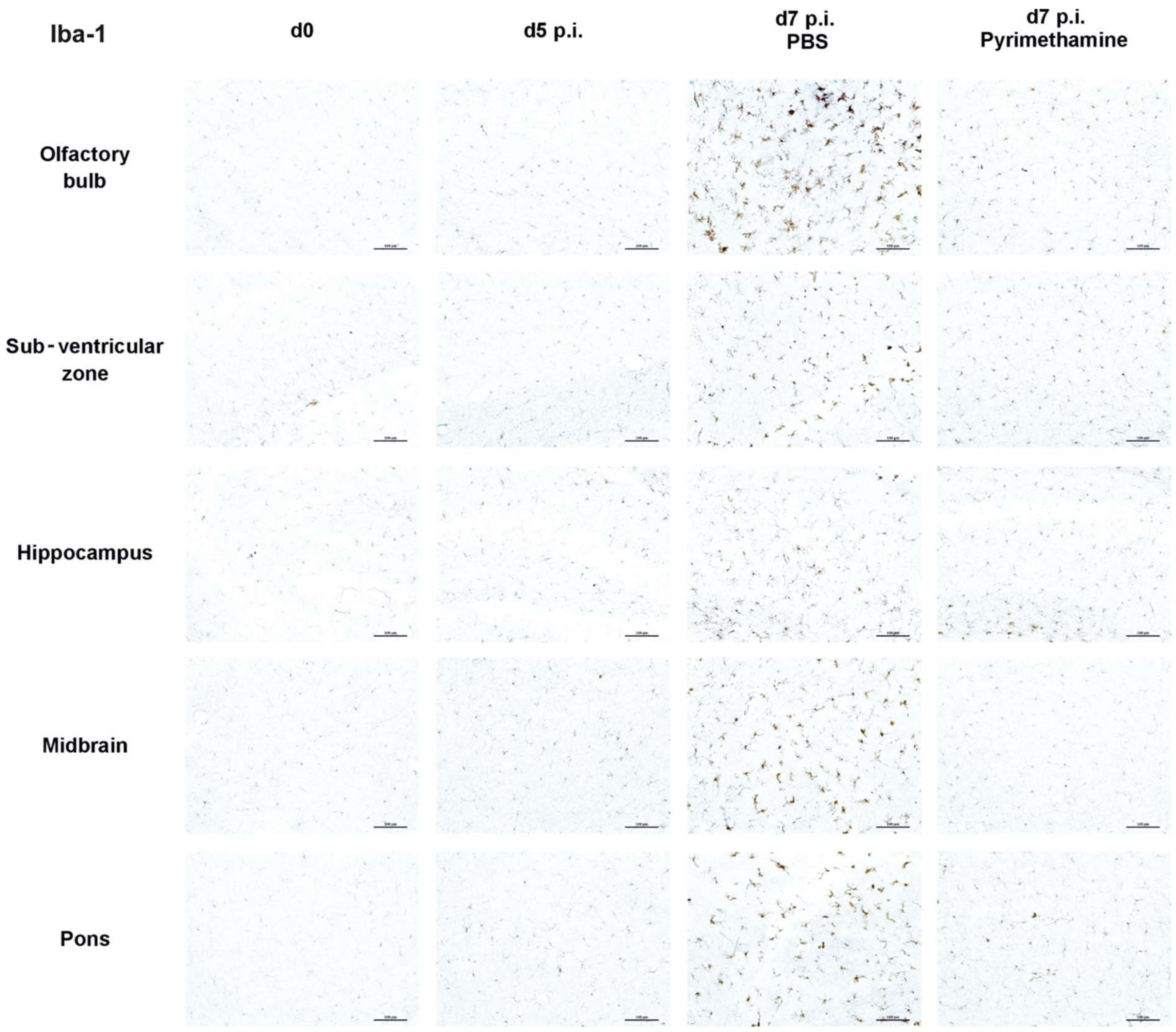

Fig. S5: Immunohistochemistry to detect activation of microglia over the course of *Plasmodium berghei* infection and after pyrimethamine treatment. Shown are representative images of histological sections of the indicated brain regions stained with an antibody against ionized calcium-binding adaptor molecule 1 (Iba-1).

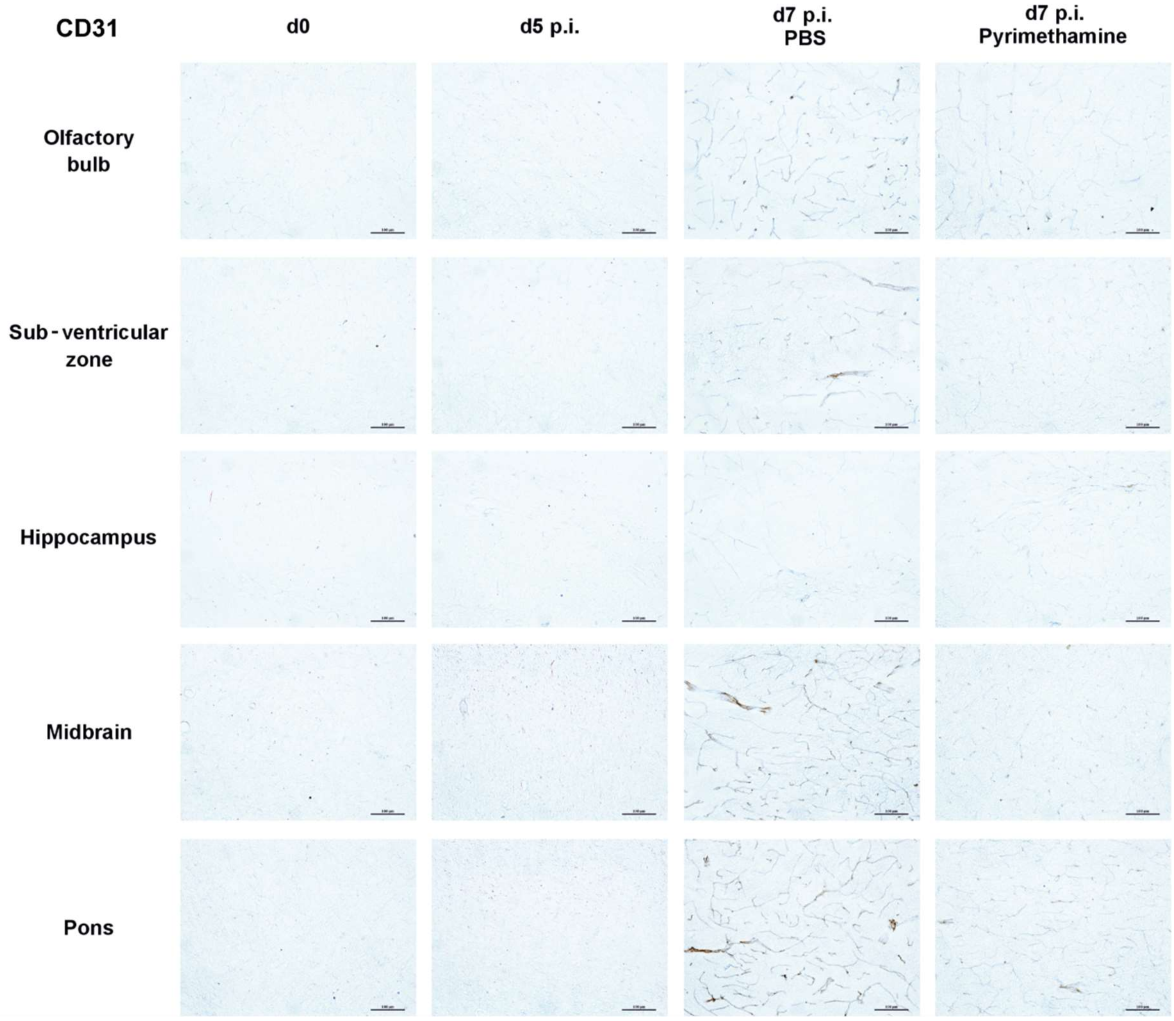

Fig. S6: Immunohistochemistry to detect activation of endothelial cells over the course of *Plasmodium berghei* infection and after pyrimethamine treatment. Shown are representative images of histological sections of the indicated brain regions stained with an antibody against CD31.

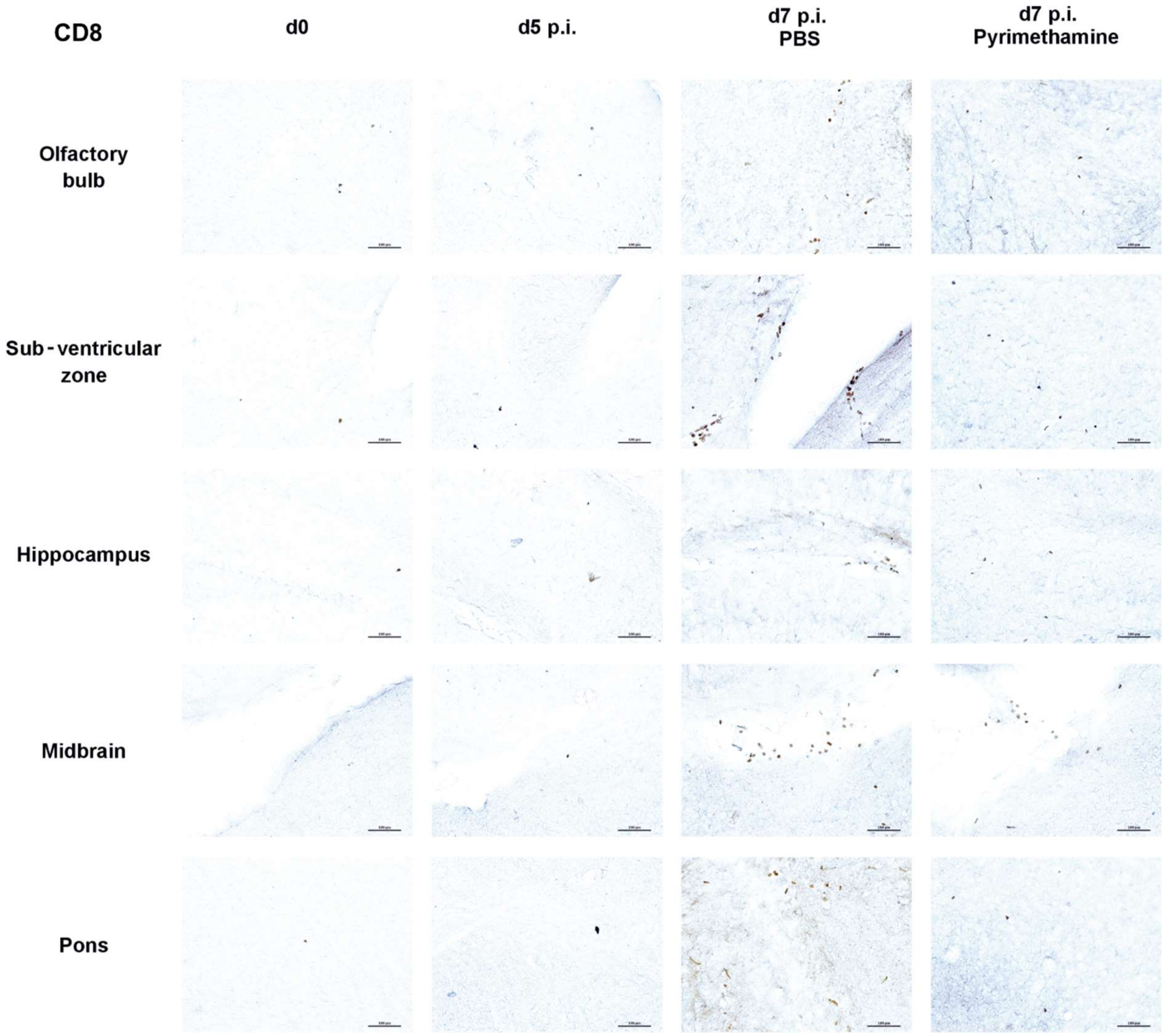

Fig. S7: Immunohistochemistry to detect increased accumulation of CD8$^+$ T cells over the course of *Plasmodium berghei* infection and after pyrimethamine treatment. Shown are representative images of histological sections of the indicated brain regions stained with an antibody against CD8.

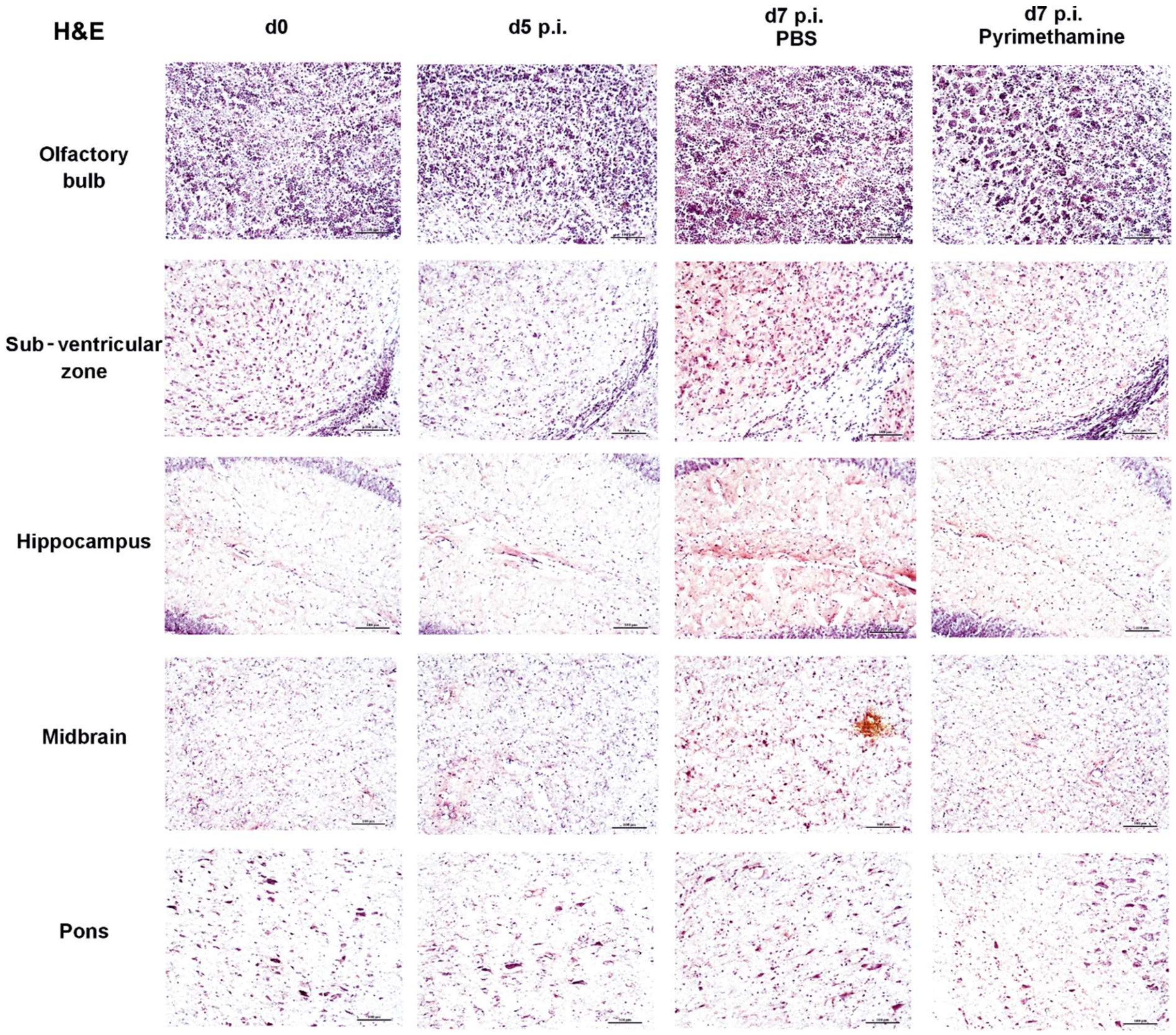

Fig. S8: Histochemistry to detect haemorrhagic lesions over the course of *Plasmodium berghei* infection and after pyrimethamine treatment. Shown are representative images of histological sections of the indicated brain regions stained with hematoxylin and eosin (H&E).

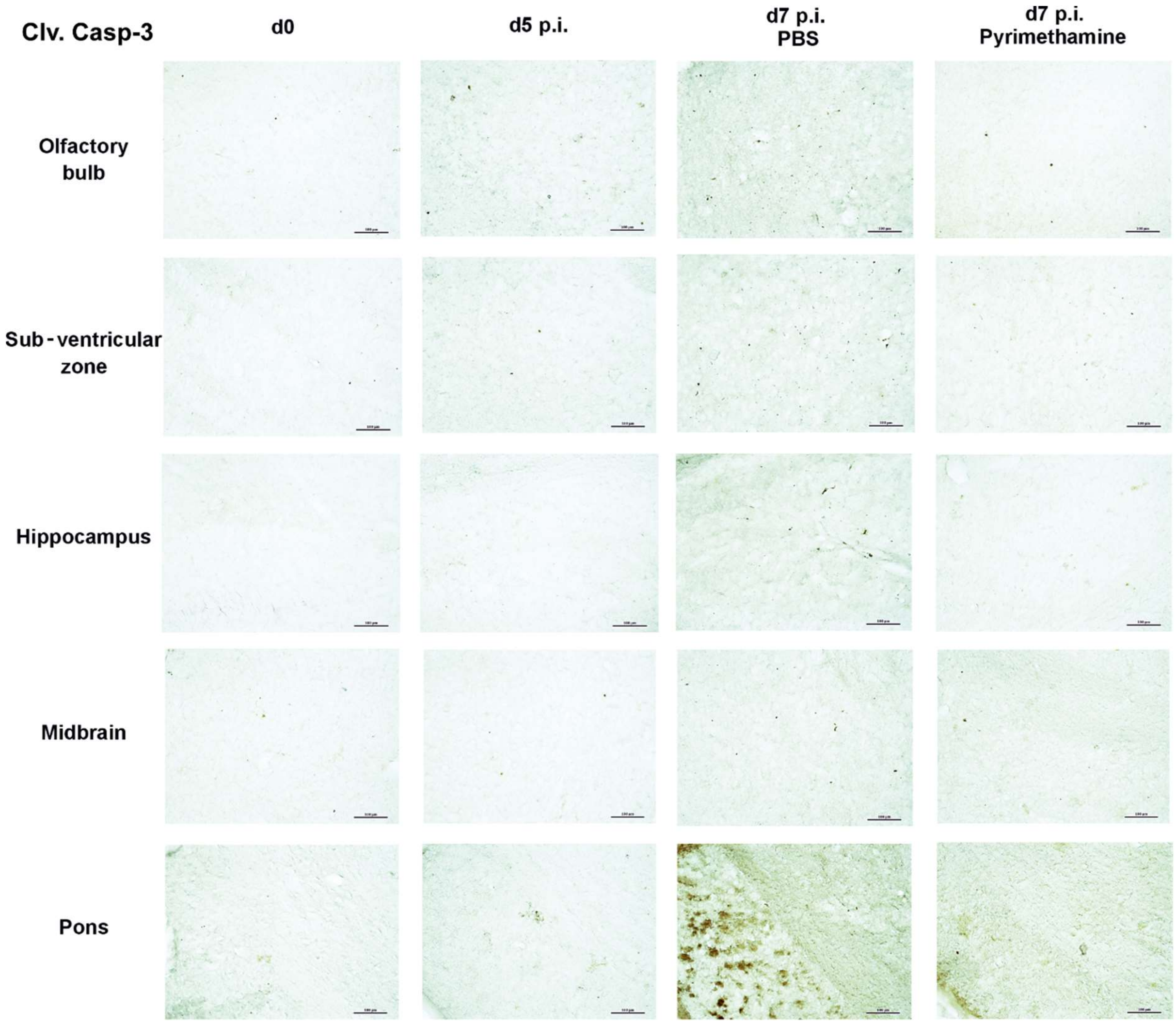

Fig. S9: Histochemistry to detect apoptotic cells over the course of *Plasmodium berghei* infection and after pyrimethamine treatment. Shown are representative images of histological sections of the indicated brain regions stained with an antibody against cleaved caspase-3.

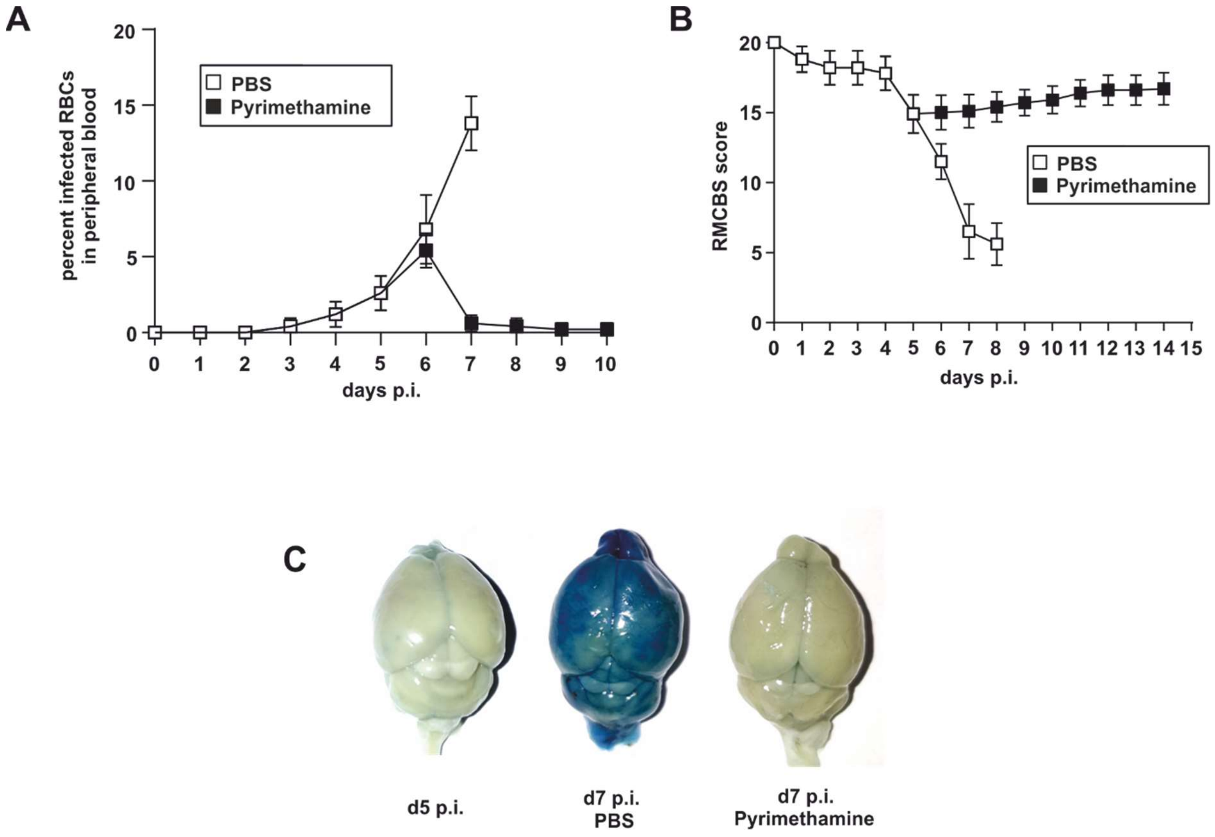

Fig. S10: **Treatment of *Plasmodium berghei* infections with pyrimethamine at the early onset prevents ECM.**

(A) Parasitemia development. Mice received a therapeutic dose of pyrimethamine on day 5 after infection. The percentage of parasitized erythrocytes in the peripheral blood was enumerated daily up to day 10 p.i. by microscopic examination of Diff. Quik-stained thin blood smears. n=5 independent animals per group. (B) The RMCBS score of appearance of neurological symptoms of ECM were monitored daily up to day 14 p.i. Data represent one of two independent experiments (n=10 independent animals per group). (C) Evans blue was injected intravenously at day 5 and day 7 p.i. The mice were euthanised 1 hour later and perfused with saline and the isolated brains were photographed. *, $p<0.05$; **, $p<0.01$; ***, $p<0.001$ (two-way ANOVA).

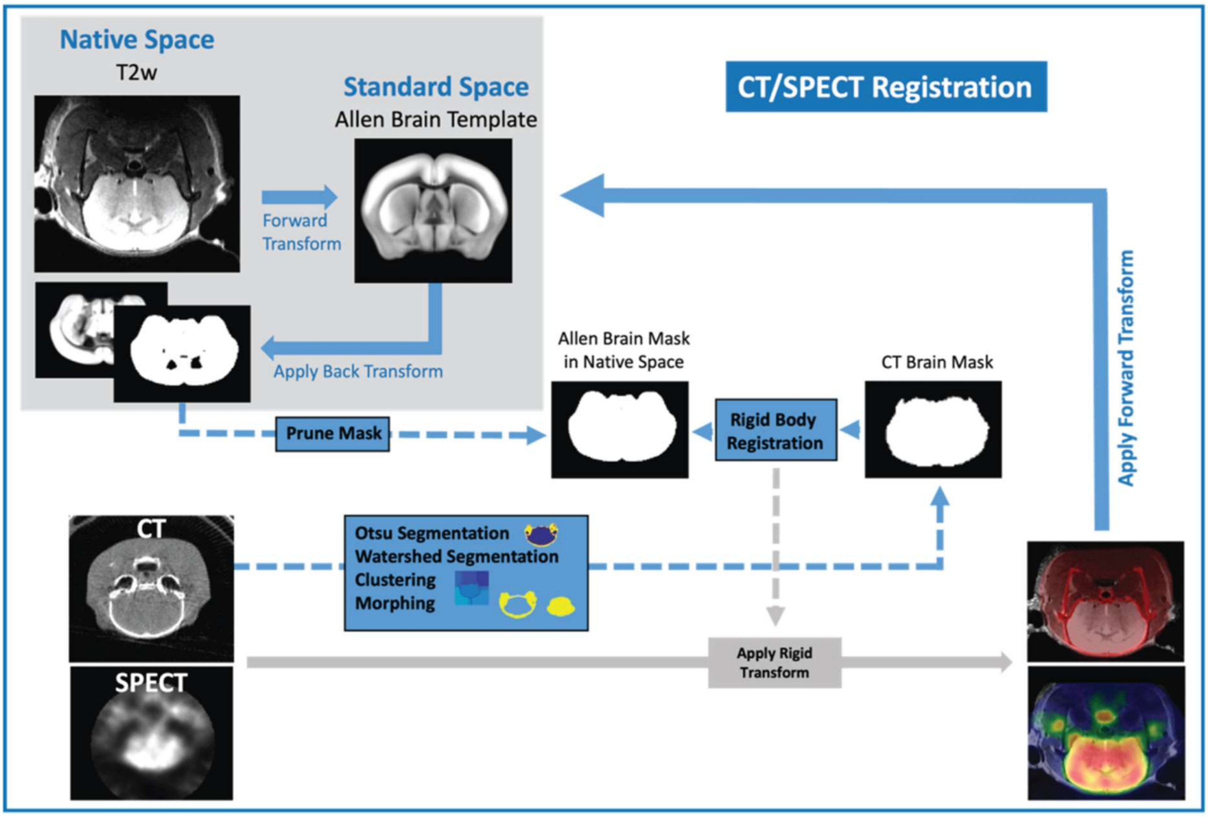

Figure S11. CT/SPECT registration pipeline.

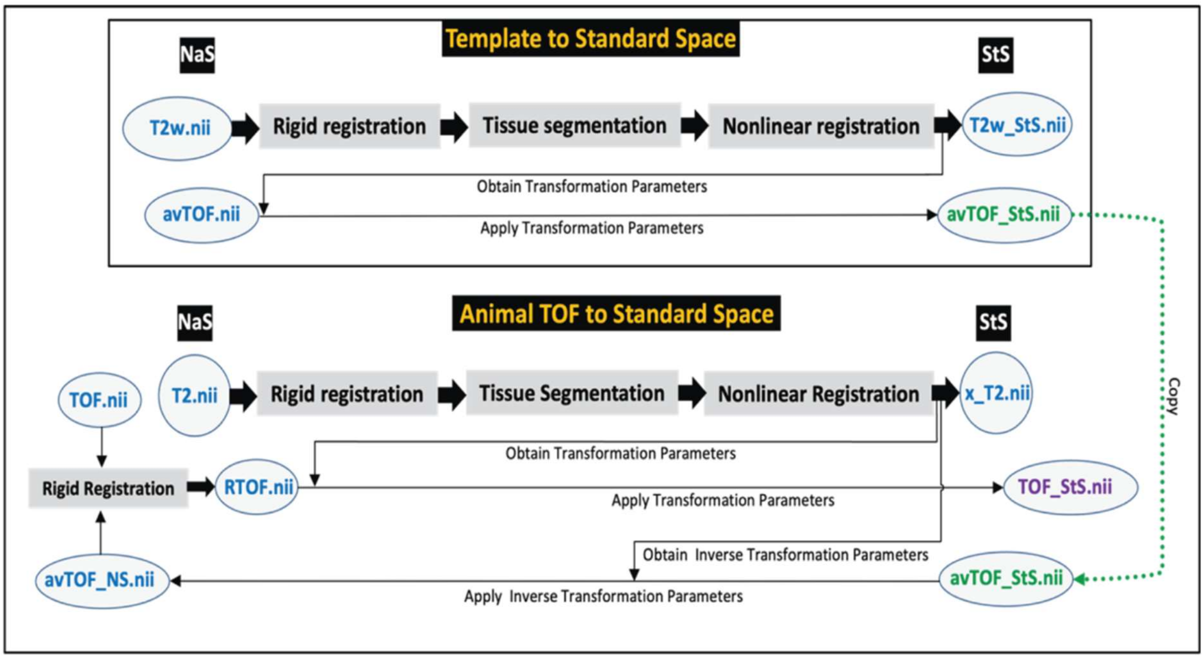

Figure S12. TOF-registration pipeline from native space (NaS) to standard space (StS, Allen brain atlas).
