# Supplementary material for: Beyond the microcirculation: sequestration of infected red blood cells and reduced flow in large draining veins in experimental cerebral malaria
Source: Nat Commun. 2024 Mar 16;15:2396. doi: 10.1038/s41467-024-46617-w (PMC10944460; doi:10.1038/s41467-024-46617-w)
Supplement: Supplementary file 3 — Reporting Summary [file 41467_2024_46617_MOESM3_ESM.pdf]

nature portfolio

Corresponding author(s): Nishanth G
Goldschmidt J

Last updated by author(s): Feb 15, 2024

Reporting Summary

Nature Portfolio wishes to improve the reproducibility of the work that we publish. This form provides structure for consistency and transparency in reporting. For further information on Nature Portfolio policies, see our Editorial Policies and the Editorial Policy Checklist.

Statistics

For all statistical analyses, confirm that the following items are present in the figure legend, table legend, main text, or Methods section.

n/a	Confirmed	
☐	☒	The exact sample size (*n*) for each experimental group/condition, given as a discrete number and unit of measurement
☐	☒	A statement on whether measurements were taken from distinct samples or whether the same sample was measured repeatedly
☐	☒	The statistical test(s) used AND whether they are one- or two-sided<br>*Only common tests should be described solely by name; describe more complex techniques in the Methods section.*
☒	☐	A description of all covariates tested
☒	☐	A description of any assumptions or corrections, such as tests of normality and adjustment for multiple comparisons
☐	☒	A full description of the statistical parameters including central tendency (e.g. means) or other basic estimates (e.g. regression coefficient) AND variation (e.g. standard deviation) or associated estimates of uncertainty (e.g. confidence intervals)
☐	☒	For null hypothesis testing, the test statistic (e.g. *F*, *t*, *r*) with confidence intervals, effect sizes, degrees of freedom and *P* value noted<br>*Give P values as exact values whenever suitable.*
☒	☐	For Bayesian analysis, information on the choice of priors and Markov chain Monte Carlo settings
☒	☐	For hierarchical and complex designs, identification of the appropriate level for tests and full reporting of outcomes
☒	☐	Estimates of effect sizes (e.g. Cohen's *d*, Pearson's *r*), indicating how they were calculated

Our web collection on statistics for biologists contains articles on many of the points above.

Software and code

Policy information about availability of computer code

Data collection	CT scan- InvivoScope 1.43<br>SPECT scan- HiSPECT<br>3D HisTech slide viewer
Data analysis	Microsoft Excel<br>GraphPadPrism 5<br>Adobe Photoshop (version 24)<br>MATLAB version R2017b<br>CT/SPECT/MRI- OsiriX software (version 5.9.1)<br>IHC-Image J software

For manuscripts utilizing custom algorithms or software that are central to the research but not yet described in published literature, software must be made available to editors and reviewers. We strongly encourage code deposition in a community repository (e.g. GitHub). See the Nature Portfolio guidelines for submitting code & software for further information.

Data

Policy information about availability of data

All manuscripts must include a data availability statement. This statement should provide the following information, where applicable:

- Accession codes, unique identifiers, or web links for publicly available datasets
- A description of any restrictions on data availability
- For clinical datasets or third party data, please ensure that the statement adheres to our policy

All data associated with this study are available in the article and the supplementary infor-mation. The source data are provided as a Source Data file. Uncropped images are available at .... Source data are provided with this paper.

Research involving human participants, their data, or biological material

Policy information about studies with human participants or human data. See also policy information about sex, gender (identity/presentation), and sexual orientation and race, ethnicity and racism.

Reporting on sex and gender	N/A
Reporting on race, ethnicity, or other socially relevant groupings	N/A
Population characteristics	N/A
Recruitment	N/A
Ethics oversight	N/A

Note that full information on the approval of the study protocol must also be provided in the manuscript.

Field-specific reporting

Please select the one below that is the best fit for your research. If you are not sure, read the appropriate sections before making your selection.

☒ Life sciences ☐ Behavioural & social sciences ☐ Ecological, evolutionary & environmental sciences

For a reference copy of the document with all sections, see nature.com/documents/nr-reporting-summary-flat.pdf

Life sciences study design

All studies must disclose on these points even when the disclosure is negative.

Sample size	A minimum of 5 mice per group were analyzed for SPECT/CT imaging and MRI. This number was sufficient to yield a high enough number of events to show statistical differences between the different experimental groups and is within the range of comparable invivo imaging studies in the field. Exact numbers are provided in figure legends. For histology and IHC 3 biological replicates were used. For qPCR a minimum of three technical replicates from 4 different mice per condition were performed. There is a vast literature describing cerebral malaria experiments in mice including the assessment of inflammation parameters, cerebral pathogenesis, protection phenotype etc. Here is the list of some of the relevant references provided in the study: Pamplonaetal.,NatMed 2007(PMID:17496899); Bapistaetal.,InfectImmun2010(PMID:20605973);Ferreiraetal.,Cell2011(PMID:21529713);Linetal.,JExpMed 2015(PMID:25941254);Strangwardetal.,PLoSPathog2017(PMID:28273147);Nizetal.,NatCommun2017(PMID:27225796);Raulfetal., CellRep2019(PMID:31269448).Therefore, the number of mice required for our experiments were decided based on the available literature.
Data exclusions	No data were excluded from analysis
Replication	All results provided in this study were reproduced with a minimum of 3 replicates.
Randomization	Random
Blinding	Blinding was not required in any experiments as phenotypes were clear

Reporting for specific materials, systems and methods

We require information from authors about some types of materials, experimental systems and methods used in many studies. Here, indicate whether each material, system or method listed is relevant to your study. If you are not sure if a list item applies to your research, read the appropriate section before selecting a response.

Materials & experimental systems

n/a	Involved in the study
☐	☒ Antibodies
☒	☐ Eukaryotic cell lines
☒	☐ Palaeontology and archaeology
☐	☒ Animals and other organisms
☒	☐ Clinical data
☒	☐ Dual use research of concern
☒	☐ Plants

Methods

n/a	Involved in the study
☒	☐ ChIP-seq
☒	☐ Flow cytometry
☐	☒ MRI-based neuroimaging

Antibodies

Antibodies used

Primary antibodies:
GFAP (rabbit Polyclonal, 1:10000, #z0334, Dako Denmark AS, Denmark)-
Ionized calcium-binding adapter molecule 1 (Iba1; rabbit polyclonal, 1:2000, #019-19741, Wako Chemicals GmbH, Germany)-
cleaved caspase-3 (Casp3; rabbit, 1:500, 882 #9661, Cell Signalling Technology Europe B.V., Germany)
CD31 (rat, 1:2000, #550274, BD Biosciences, Germany)
CD8 (rat, 1:1000, #14-0808-82, eBioscience, Affymetrix Inc., 37 884 USA)-
DCX (goat, 1:1000, #sc-8066, Santa Cruz Biotechnology, USA
BrdU (rat, 1:2000, #ab6326, Abcam, United Kingdom)-
biotinylated secondary antibodies :
anti-rabbit: 885 1:200, #111-065-144, Dianova, Germany
anti-rat: 1:200, #712-065-153, Dianova, Germany
Other secondary antibodies:
anti-goat, Alexa-488 , 1:200, #A11055, Invitrogen by Thermo Fischer Scientific, USA
anti-rat, Cy3, 1:200, #712-165-153, Dianova, Germany

Validation

Primary antibodies:
GFAP (rabbit Polyclonal, 1:10000, #z0334, Dako Denmark AS, Denmark)- https://www.agilent.com/en/product/immunohistochemistry/antibodies-controls/primary-antibodies/glial-fibrillary-acidic-protein-%28concentrate%29-76683
Ionized calcium-binding adapter molecule 1 (Iba1; rabbit polyclonal, 1:2000, #019-19741, Wako Chemicals GmbH, Germany)- https://labchem-wako.fujifilm.com/us/product/detail/W01W0101-1974.html
cleaved caspase-3 (Casp3; rabbit, 1:500, 882 #9661, Cell Signalling Technology Europe B.V., Germany)- https://www.cellsignal.com/products/primary-antibodies/cleaved-caspase-3-asp175-antibody/9661
CD31 (rat, 1:2000, #550274, BD Biosciences, Germany)- https://www.bdbiosciences.com/en-de/products/reagents/flow-cytometry-reagents/research-reagents/single-color-antibodies-ruo/purified-rat-anti-mouse-cd31.550274
CD8 (rat, 1:1000, #14-0808-82, eBioscience, Affymetrix Inc., 37 884 USA)- https://www.thermofisher.com/antibody/product/CD8a-Antibody-clone-4SM15-Monoclonal/14-0808-82
DCX (goat, 1:1000, #sc-8066, Santa Cruz Biotechnology, USA) - https://www.scbt.com/p/doublecortin-antibody-c-18
BrdU (rat, 1:2000, #ab6326, Abcam, United Kingdom)- https://www.abcam.com/en-de/products/primary-antibodies/brdu-antibody-bu1-75-icr1-proliferation-marker-ab6326
Secondary antibodies:
biotinylated secondary antibodies
anti-rabbit: 885 1:200, #111-065-144, Dianova, Germany-https://www.dianova.com/en/shop/111-065-144-goat-igg-anti-rabbit-igg-hl-biotin-minx-humsrt/
anti-rat: 1:200, #712-065-153, Dianova, Germany- https://www.dianova.com/downloads/Jackson/712-065-153.pdf
Other secondary antibodies
anti-goat, 1:200, #A11055, Invitrogen by Thermo Fischer Scientific, USA- https://www.thermofisher.com/antibody/product/Donkey-anti-Goat-IgG-H-L-Cross-Adsorbed-Secondary-Antibody-Polyclonal/A-11055
anti-rat, 1:200, #712-165-153, Dianova, Germany-https://www.dianova.com/downloads/Jackson/712-165-153.pdf

Animals and other research organisms

Policy information about studies involving animals; ARRIVE guidelines recommended for reporting animal research, and Sex and Gender in Research

Laboratory animals

C57BL/6 WT mice: 12.7 weeks mean
C57BL/6 prf-/- mice: 15.8 weeks mean
BALB/c mice: 11 weeks mean

Wild animals

No wild animals were used in this study

Reporting on sex

Both male and female mice were used in this study. Sex of each mouse used is mentioned in methods sections.

Field-collected samples

This study did not include field-collected samples

Ethics oversight | All animal experiments were in compliance with the German Animal Welfare Act in a protocol approved by the state authorities (Landesverwaltungsamt Sachsen Anhalt).

Note that full information on the approval of the study protocol must also be provided in the manuscript.

Plants

Seed stocks | *Report on the source of all seed stocks or other plant material used. If applicable, state the seed stock centre and catalogue number. If plant specimens were collected from the field, describe the collection location, date and sampling procedures.*

Novel plant genotypes | *Describe the methods by which all novel plant genotypes were produced. This includes those generated by transgenic approaches, gene editing, chemical/radiation-based mutagenesis and hybridization. For transgenic lines, describe the transformation method, the number of independent lines analyzed and the generation upon which experiments were performed. For gene-edited lines, describe the editor used, the endogenous sequence targeted for editing, the targeting guide RNA sequence (if applicable) and how the editor was applied.*

Authentication | *Describe any authentication procedures for each seed stock used or novel genotype generated. Describe any experiments used to assess the effect of a mutation and, where applicable, how potential secondary effects (e.g. second site T-DNA insertions, mosiacism, off-target gene editing) were examined.*

Magnetic resonance imaging

Experimental design

Design type | Longitudinal anatomical and angiographic measurements

Design specifications | Three mouse strains were imaged, C57Bl6 wt, C57Bl 6 prf -/- and BALB/c. Mice were imaged before infection, at day 5 p.i. and at day 7 p.i.

Behavioral performance measures | No behavioral measures

Acquisition

Imaging type(s) | Structural MRI and MR angiography

Field strength | 9.4

Sequence & imaging parameters | Angiography with 2D MR-TOF with flow compensation (TR/TE: 3 ms/12 ms, in 80 slices with a slice thickness of 0.3 mm, slice overlaps of 0.1 mm and an in-plane-resolution of 0.08 x 0.08 mm, measured in caudo-rostral direction. Flip Angle was set to 70°, saturation slice, of 3mm thickness, was positioned with a 1mm gap towards the caudal side of the brain); Anatomical Reference with a 2D T2 TurboRARE (TR/TE = 4200 ms / 18.5 ms, 2 averages, FoV of 25.6 x 25.6 mm, RARE-Factor 8, slice thickness of 0.2mm and in-plane resolution of 0.1 x 0.1 mm)

Area of acquisition | Whole brain imaging

Diffusion MRI ☐ Used ☒ Not used

Preprocessing

Preprocessing software | Paravivion 6.0.1

Normalization | Data from ROI analysis were normalized to mean signal intensity at d0 in each group

Normalization template | N/A

Noise and artifact removal | No noise/ artifacti removal was performed

Volume censoring | Censoring was not performed

Statistical modeling & inference

Model type and settings | N/A

Effect(s) tested | N/A

Specify type of analysis: ☐ Whole brain ☒ ROI-based ☐ Both

Anatomical location(s) | ROI-based analysis covering both RRVs and the rotsral SSS

Statistic type for inference<br>(See Eklund et al. 2016)	Unpaired hetereoscedastic t-test.
Correction	No corrections

Models & analysis

n/a	Involved in the study
☒	☐ Functional and/or effective connectivity
☒	☐ Graph analysis
☒	☐ Multivariate modeling or predictive analysis
